# PROGame: A process framework for serious game development for motor rehabilitation therapy

**DOI:** 10.1371/journal.pone.0197383

**Published:** 2018-05-16

**Authors:** Esperança Amengual Alcover, Antoni Jaume-i-Capó, Biel Moyà-Alcover

**Affiliations:** Department of Mathematics and Computer Science, University of the Balearic Islands, Palma de Mallorca, Spain; IRCCS E. Medea, ITALY

## Abstract

Serious game development for rehabilitation therapy is becoming increasingly popular because of the motivational advantages that these types of applications provide. Consequently, the need for a common process framework for this category of software development has become increasingly evident. The goal is to guarantee that products are developed and validated by following a coherent and systematic method that leads to high-quality serious games. This paper introduces a new process framework for the development of serious games for motor rehabilitation therapy. We introduce the new model and demonstrate its application for the development of a serious game for the improvement of the balance and postural control of adults with cerebral palsy. The development of this application has been facilitated by two technological transfer contracts and is being exploited by two different organizations. According to clinical measurements, patients using the application improved from high fall risk to moderate fall risk. We believe that our development strategy can be useful not only for motor rehabilitation therapy, but also for the development of serious games in many other rehabilitation areas.

## Introduction

Serious games are computer games designed for a primary purpose other than pure entertainment. In various areas (e.g., defense, education, scientific exploration, healthcare, emergency management, city planning, engineering, and politics), the ultimate purpose of a serious game is to allow users to reach a specific goal in an entertaining and engaging manner through the experience of playing the game.

In the healthcare field, serious games are largely focused on treatment, recovery, and rehabilitation. In this work, we consider the development of serious games for motor rehabilitation therapy, an area where these games are considered to be a highly promising solution [1] because it has been demonstrated that they help to motivate patients during therapy sessions [2]. This motivation is particularly important in long-term rehabilitation for maintaining motor abilities, where demotivation is frequent in chronic patients because therapy typically consists of repetitive and intensive activities that become boring after hundreds of sessions [3–6].

In this paper, we present a disciplined and repeatable process framework that supports the development of serious games for motor rehabilitation. Our method has been clinically validated. The remainder of this paper is structured as follows. Firstly, we discuss some desirable features of motor rehabilitation serious games that we have encountered in most serious game projects and introduce the theoretical basis of serious games with the requirements that a process framework for the development of serious games for rehabilitation should satisfy. Secondly, we describe PROGame, a novel process framework for the development of serious games for motor rehabilitation. Thirdly, we present a validation of this framework through the development of a specific serious game. Finally, we present our conclusions and discuss future research.

## Desirable features for motor rehabilitation serious games

Before we define a new process framework for serious game development, we discuss a few important aspects that we have encountered in most serious game projects for motor rehabilitation therapy.

Burke et al. [4] identified two principles of game design theory that have particular relevance to rehabilitation: meaningful play (the relationship between player interactions and system reactions) and challenge (maintaining an optimal difficulty is important to maintaining player engagement). Jaume-i-Capó et al. [7] enumerated the desirable features of rehabilitation serious games:

1. Selecting an existing interaction mechanism. A serious game should not develop a new rehabilitation therapy because this would make it difficult to validate both the therapy and the serious game. It is more suitable to use an existing therapy as the mechanism of interaction with the serious game. This means that if the patients want to play, they must perform the therapy.
2. Defining an interaction model adapted to patient capabilities. The interaction model must be defined according to the selected therapy and patient physical capabilities. For the therapy, one should consider the part of the body where the therapy is applied and the resolution of the therapy measurements among other factors. For patient capabilities, it is important to consider that patients may find it difficult to properly hold physical devices.
3. Incremental development. Our experience with this type of project has shown us that to ensure all objectives are met, we must follow an incremental development process that supports communication, an activity that we consider crucial because technical engineering language is completely different from therapy specialist language. Furthermore, incremental development accommodates validation of the system, which is another important activity.
4. Specialist validation. Specialists must validate that every increment facilitates the performance of the selected therapy. Otherwise, the final game would not have the desired therapeutic effect.
5. Clinical evaluation. The serious game must be clinically validated to determine the effects of the physiotherapy treatment and compare them to the effects of standard therapy methods to determine if the serious game is appropriate for the motor rehabilitation goal. Specialist validation must be precise because clinical evaluation is expensive in terms of resources and time, and must be approved by an ethical committee.

## A serious game development process: Theoretical basis

As defined in well-known software engineering fundamentals, a software process is a set of related activities that lead to the production of a software product [8,9]. There are many different software processes, but all must include the four basic process activities [10]: (1) specification, (2) design and implementation, (3) validation, and (4) evolution. These activities are organized differently in different development processes.

Given the current importance of serious game development and based on the software engineering fundamentals [10–12] we believe that a systematic and organized approach to serious game development is necessary to produce high-quality products. To the best of our knowledge, there are no existing process frameworks for serious game development for motor rehabilitation therapy. However, some relevant design issues have been published in the literature [7,13] which are essential to provide suitable solutions for specific development efforts. At this point, we consider it important to highlight that the goal of this work is to create a framework that goes beyond these design considerations to handle other dimensions of the development process. The interdisciplinary nature of serious game development requires a holistic and validated framework that provides guidance on system development tasks with a wider focus than the simplistic view of general design issues. In the following section, we introduce PROGame, a framework for the development of serious games for motor rehabilitation therapy. Drawing on our experience in the development of serious games [14,15], as well as software process assessment and improvement [16–19], we combine three different development approaches.

First, the need for an agile model led us to use Scrum as a reference framework [20] for managing and controlling iterative work at the project level.

Second, PROGame is based on the web application development model introduced in [21]. We decided to use this model as a reference for our framework because we believe that serious game development requirements have some similarities to web application requirements [22].

Finally, we believe that the implementation of a serious game for motor rehabilitation is similar to a clinical trial involving new drugs [23].

### Scrum for agile project management

A development project for a serious game for motor rehabilitation therapy involves a multidisciplinary team with diverse stakeholders. As discussed before, support for continuous communications between developers and therapy specialists, each with their own technical language, is an important challenge to overcome. An incremental agile method is promising not only for managing communication, but also for supporting incremental development that accommodates validation activities. It is particularly important to test critical nonfunctional requirements, such as the safety of interaction mechanisms.

As highlighted in [10], agile approaches are particularly successful for development projects where there is a clear commitment from the customer to be involved in the development process. Agile approaches work well in these situations because it is possible to have continuous communication between stakeholders.

The choice of Scrum in particular was motivated by the fact that it focuses on supporting agile project management, which we consider to be an essential activity for managing and controlling the aforementioned basic process activities.

### Requirement similarities between web applications and serious games

Web engineering is considered to be a specialization of software engineering by prestigious software engineering specialists and project managers, such as Roger Pressman, Tom DeMarco, and Watts Humphrey [24]. Following from this concept, the basic principles of web engineering have been described similarly to those of software engineering: clearly defined requirements, systematic development, and careful project management.

From the same viewpoint as the authors above, we have considered that existing software development techniques can be adapted to develop serious games in the same way that they have been adapted to the development of web applications [22]. Both web applications and serious games differ from conventional software systems in various ways (e.g., multidisciplinarity of stakeholders, significance of quality aspects, quality of user interfaces, and developer inexperience).

### Requirement similarities between a clinical trial for new drugs and the development of serious game for motor rehabilitation

We have observed certain similarities between the development of a serious game for motor rehabilitation [6,14,15,25,26] and a clinical trial for new drugs [23]. In both cases, the process requires a set of sequential phases, where each phase attempts to meet or define some objective.

In drug development, there are five necessary phases to prove that a drug is suitable for medical treatment (see Table 1).

**Table 1:** Phases of a clinical trial for drug development.

Phase	Drug-development
Phase 0 – Ensure efficacy	The drug is tested on non-human subjects to determine its efficacy.
Phase I – Ensure safety	The drug is tested on healthy volunteers to determine its safety.
Phase II – Test on patients	The drug is tested on patients to determine its efficacy on humans.
Phase III – Test therapeutic effects	The drug is tested on patients to determine its therapeutic effects.
Phase IV – Determine long-term effects	The drug is monitored during public use to determine its long-term effects.

We propose a five-phase process for developing serious games for motor rehabilitation, using a clinical trial as a reference:

1. Therapy selection: The development team studies existing validated therapies for clinical goals, as well as the capabilities of patients, and selects an appropriate therapy. This allows us to ensure the efficacy of the therapy before implementing it in a serious game. We compare this phase to Phase 0 in drug development because the therapy selection phase also ensures the efficacy of the treatment before applying it to patients.
2. Interaction mechanism: Accounting for existing technology, the engineering team designs an interaction mechanism to capture the therapy in a serious game. In this phase, the therapists must ensure that the interaction mechanism is safe and that it matches the motor capabilities of patients. For example, if the therapy requires arm movement, but the user cannot hold a device, cameras and computer vision methods could be selected as interaction mechanisms. The interaction mechanism is validated by physiotherapists and tested on real patients to verify that the interaction mechanism facilitates the therapy in a safe manner. We compare this to Phase 1 in drug development because the interaction mechanism phase also ensures that the treatment is safe for patients.
3. Interaction elements: We must ensure that the patient is performing the therapy correctly. The team designs interaction elements that force patients to perform the therapy correctly. In this phase, therapists must ensure that the interaction elements force the patients to perform the selected therapy. Interaction elements are validated by physiotherapists and tested on real patients to verify that when they interact with the elements, they are performing the therapy in an effective manner. We compare this to Phase 2 in drug development because the interaction elements phase also ensures that the treatment is effective for patients.
4. Serious game: Following the principles of game design theory, the development team designs a serious game to encourage the user to perform the therapy regularly to achieve a therapeutic effect. In this phase, therapists must ensure that playing the serious game helps patients to achieve the desired therapeutic effect. The serious game is validated by physiotherapists and tested on real patients to verify that when patients play the serious game, they are achieving the desired therapeutic effect. We compare this to Phase 3 in drug development because the serious game phase also ensures that treatment achieves the desired therapeutic effect.
5. Clinical study: The development team verifies that the serious game has the same effects as the selected therapy to determine its long-term effects. We compare this to Phase 4 in drug-development because the clinical study phase also ensures that treatment has the desired long-term effects.

## PROGame: A process framework for serious game development

**Fig 1:**
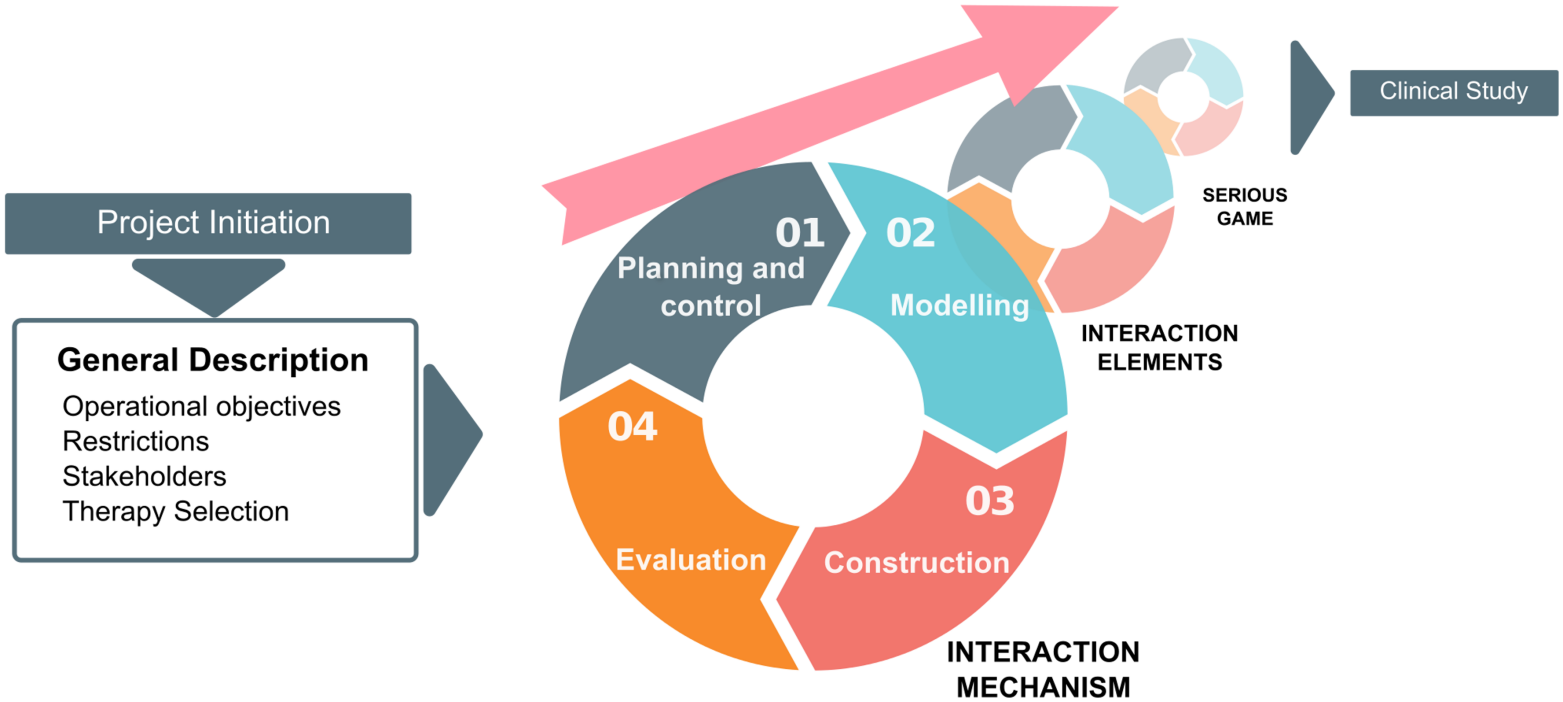
Process flow for serious game development.

Considering the realities and specific characteristics of serious game development described in the previous sections, we believe that PROGame activities must be repeated to design and deliver an incremental solution. Fig 1 illustrates the process flow of development activities.

As shown in Fig 1, the serious game development framework follows an iterative process flow structured in two dimensions, which are described in detail below.

### First dimension activities

The first dimension includes a project initiation activity, an iterative flow composed of four basic development activities, and a final clinical study. The structure of this dimension has been designed based on the three aforementioned approaches: Scrum, the web application development model, and a clinical trial:

- Scrum is the basis of the first activity of the framework. The project initiation activity has been adapted from the sprint 0 phase, which is the initial Scrum phase for starting a new project.
- The basic development activities, which are organized as an iterative flow, have been adapted from the web application development model.
- Phase 0 of the clinical trial has been included as a therapy selection task within the first activity. The framework also considers a clinical study as the final activity to replicate Phase IV of the clinical trial fundamentals.

#### Project initiation

The serious game development process begins with the *project initiation* activity. This activity serves as the entry point for the process flow. It is where engineers and therapists consider fundamental questions regarding the game and its context, and determine patient needs. The activity begins by identifying the context, operational objectives, and restrictions for the serious game as a whole. Then, a more detailed specification of the game’s requirements and constraints is created. This information is compiled in a general description that is used as the basis for identifying the therapy to be implemented and for developing a macroscopic schedule for the three subsequent iterations.

More specifically, the project initiation activity includes the following tasks:

- Identify the need for a serious game. Describe the need for a serious game and consider the possible economic, technical, operational, and legal restrictions that could affect development.
- Identify stakeholders and user categories. Identify exactly who is the customer for the serious game and which people can serve as experts and representative end users. Identify how many different types of users will interact with the serious game, as well as any special needs and required functionality for each user category.
- Identify the game functionality and constraints. Define the functional requirements of the game. Engineers and physiotherapists prepare a list containing the information that the game must manage and the functionality that it must provide. Possible constraints are also identified.
- Select the therapy for the serious game. Considering the game functionality, patient capabilities, and constraints, select a validated therapy to transfer into the serious game.

#### Iterative flow

The iterative flow in the first dimension of the model is structured into the four basic development activities: planning and control, modeling, construction, and evaluation.

The main goal of the *planning and control* activity is to perform incremental project management for the development of the serious game to minimize the effort and time spent on managing the complexity of the increment to be deployed. This activity includes three tasks: planning, scheduling, and tracking. The goal of *planning* is to determine the tasks to perform during the increment by identifying the end products of the increment and the people who will do the work:

- Determine the tasks to perform during the increment. Refine the modeling, construction, and validation tasks for the increment.
- Identify products. The number of products developed should be the minimum necessary amount to facilitate the subsequent task.

*Scheduling* is the task that allocates the estimated effort for specific tasks across the planned schedule for building an increment. The main goal is to create a list of tasks and identify any interdependencies. It is also important to identify critical tasks that should be monitored to identify possible deviations as soon as possible. The goal of the *tracking* task is to measure the development progress. If the development team has taken the time to construct a detailed work schedule for the increment, progress can be tracked by determining how many work tasks have been completed, how many work products have been produced and reviewed, and how much confidence individual team members have in the increment completion date.

The purpose of the *modeling* activity is the development of models that help the development team to better understand the requirements and design of the serious game. The starting point is the information obtained in the project initiation activity (if on the first iteration) or the requirement information obtained from previous iterations. The complexity of the modeling activity can be managed by considering different perspectives for modeling distinct aspects, such as content or interaction with the user interface. More specifically, the tasks to consider during modeling are the following:

- Refine functional requirements. Detail the user requirements for the increment.
- Design application contents. Analyze the functional requirements to create a content model with information entities that the user will create or modify when playing the serious game.
- Design the graphical user interface. Use prototyping to design the user interface for the increment.
- Identify and design functional components. Analyze and design the functionality of the system components to implement for the increment.

The *construction* activity produces executable software units that properly reflect the design. Construction includes the production and testing of software units that are part of an increment. It is important to emphasize that to maintain agility, the software units to be constructed are designed during the construction activity, rather than in the previous modeling activity.

The following tasks are part of the construction activity:

- Select reusable components. Consider the constraints of the technical environment, skills and knowledge of the developers, and issues regarding intellectual property to select the most suitable reusable components.
- Construct executable components. Follow standard coding principles to write code to produce executable components for the increment.
- Test executable components. Perform unit tests and correct errors.

The purpose of the *evaluation* activity is playing the serious game with the goal of finding and correcting errors before it is made available to patients. In addition to the conventional quality aspects that any software application must address, the evaluation objectives within the context of a serious game for motor rehabilitation must consider the following aspects:

- The designed interaction must be possible and safe for the motor capabilities of patients.
- The therapy must be effective.
- The game must engage the user.

#### Clinical study

**Fig 2:**
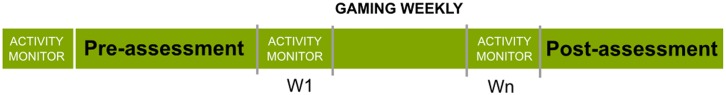
Clinical study assessments.

The *clinical study* activity aims to quantify the improvement of rehabilitation based on types of functional exercises. In order to perform a successful clinical evaluation, this activity must define the experiment, participants, and measurements according to the final goals and type of therapy. In [7], it was recommended to design the entire study through a pre-assessment and post-assessment of every measurement (see Fig 2). Optimally, the success of the clinical evaluation increases when one can include a control group and the greatest possible number of measurements [27].

### Second dimension: Incremental development

The second dimension supports the three core phases of the clinical trial previously described. These phases have been included in this dimension as three different increments: interaction mechanism, interaction elements, and serious game.

The goal of the first increment, *interaction mechanism*, is to design an interaction mechanism to capture the selected therapy while considering existing technology. It is important to ensure that the provided solution is safe for the motor capabilities of patients. For example, if the therapy requires arm movement and the patient cannot hold a device, cameras and computer vision could be used as interaction mechanisms.

During the second increment, *interaction elements*, the development team must design the interaction elements that force patients to perform the therapy correctly to ensure that the therapy is effective.

The final increment is aimed at designing a serious game to encourage the patient to perform the therapy regularly to reach a therapeutic effect. We call this increment the *serious game*.

## Framework validation

In this section, we introduce the project used to validate the new framework. We had the opportunity to apply PROGame to the development of a serious game for the improvement of balance and postural control in adults with cerebral palsy (CP) [15].

The project was supported by the Spanish Association of Cerebral Palsy (ASPACE), whose mission is comprehensive care for people with CP and related diseases. The goal of this association is to improve personal autonomy, facilitate integration into society, and improve the quality of life of people with CP by applying multidisciplinary medical rehabilitation, education, employment, and social assistance.

The goal of this project was to provide a technology-assisted therapy to improve and maintain quality of life and independence for adults with CP. Although a great deal of research has focused upon improving quality of life for children with CP, few studies have emphasized systems and methods for adults with CP.

In order to present the results of the validation of PROGame, we have divided this section into five parts: *project initiation*, *interaction mechanism*, *interactive elements*, *serious game*, and *clinical study*.

### Project initiation

Following the process flow of the proposed framework, the first task we performed was the identification of the need for a serious game, the stakeholders, and user categories, as well as the requirements of the game (operational objectives and restrictions). This information, summarized below, was used as the basis to select the therapy to be implemented by the game.

#### General description

Many children and adults with CP have poor walking abilities and manipulation skills. One of the factors that contribute to problems with gait and reaching movements is poor balance control. This is because the maintenance of stability is critical to all movements [28]. The objectives of medical intervention and physical therapy are to improve balance and postural control, prevent dependence, and preserve autonomy.

The use of a computer as a rehabilitation tool is considered as a possible alternative therapy that could help motivate patients during therapy sessions. This idea is based on the fact that the use of a computer motivates users to work in an entertaining manner.

The goal of the project is to provide a rehabilitation game that motivates users through new technologies. Specifically, the game must fulfill the following operational objectives:

- Training balance control.
- Improving coordination and trunk control.
- Encouraging cognitive and communicative aspects.
- Improving functional capabilities in daily life activities.
- Avoiding therapy abandonment.

The serious game must include the desirable features for rehabilitation, which are feedback and adaptability to user capabilities [4]. Furthermore, the new serious game should allow the inclusion of motivational elements to increase engagement. It should also include monitoring mechanisms to simplify the therapist’s work [7].

In this project, two different technical aspects are considered to be crucial.

The first technical consideration is the use of a specific interaction technology, computer vision, also known as vision-based interaction (VBI) [29]. Visual information from the performance of user actions is the preferred capture method for two reasons. First, motor rehabilitation consists of body movements that can be recorded. Second, vision capture technology is noninvasive and can be used by patients who have difficulty holding physical devices.

The second technical consideration is the use of mirror feedback. Mirror feedback consists of projecting the user onto the screen and simulating a mirror in such a way that the users can see themselves on the screen at all times. We must include this technical restriction because mirror movements and imitation learning is highly recommended for motor rehabilitation [30] and the advantages of observation and imitation for learning have been well studied [31]. For this reason, mirrors are included in motor therapy rooms because they allow patients to see themselves and verify that they are correctly performing the therapy.

In order to increase the number of therapy sessions and exercises, it is important to offer to users the possibility of performing rehabilitation at home. For this reason, we need a low-cost system. Currently, low-cost RGBD devices that can capture user movements at a frame rate of up to 30 fps are widely available.

For testing and clinical studies, users or their families signed an informed consent form [14,15]. Furthermore, we obtained the approval of ASPACE board prior to experimentation to correctly apply legislation and consider ethical issues (human dignity, confidentiality, non-discrimination, and proportionality between risks and benefits).

In order to better classify the different stakeholders and their roles within the project, we elaborate on the stakeholder specifications in Table 2.

**Table 2:** Stakeholder specifications.

Type	Role
Hospitals and rehabilitation centers	Provide facilities, specialists, and patients
Physicians/Physiotherapists	Game design Validation of rehabilitation therapy
Patients	Play the serious game
Families	Legal proxies (if needed)
Engineers	Serious game development
Designers	Creative activities (art, animation, etc.)
Ethical committee	Clinical study authorization

**Fig 3:**
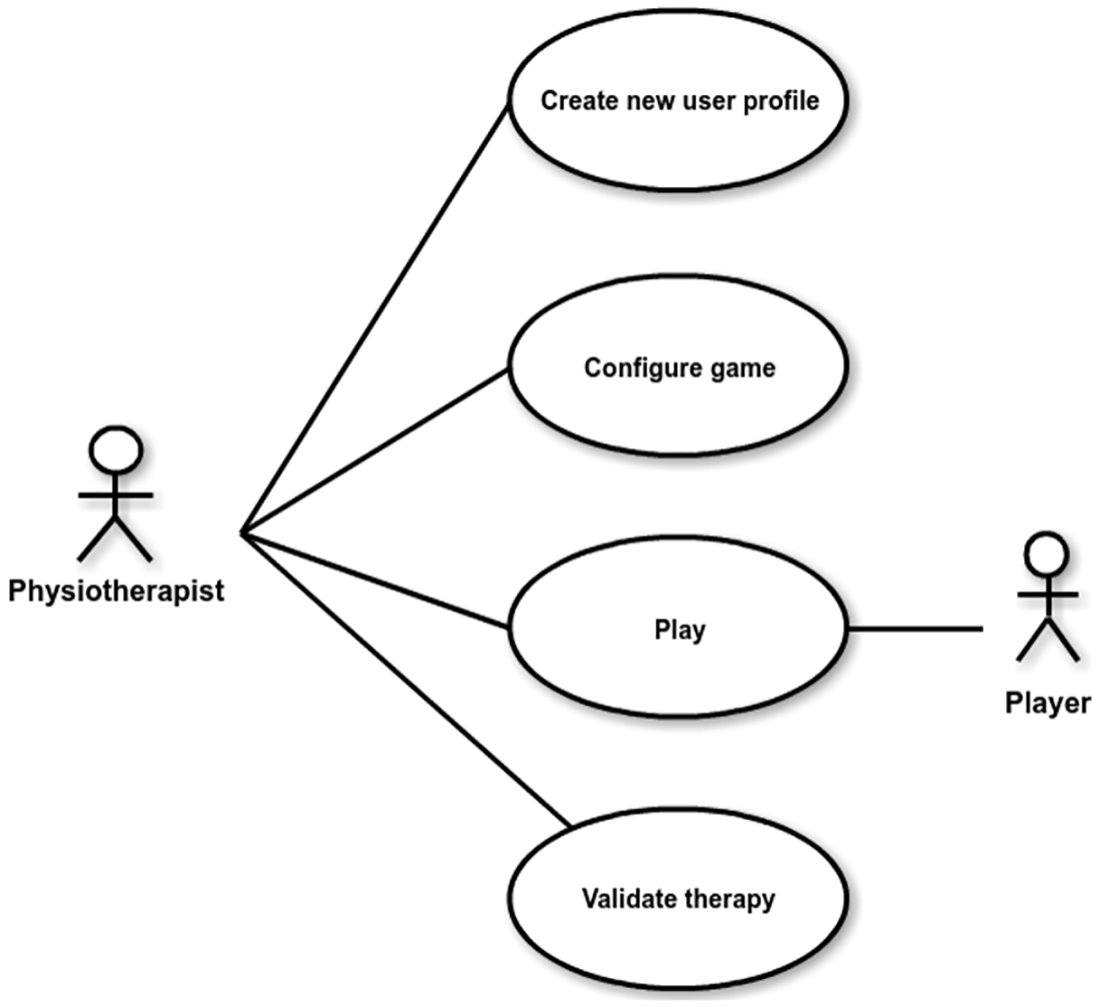
Overview of the functionality of our serious game.

Once the different stakeholders are identified, engineers and physiotherapists work together to define the requirements of the game. Fig 3 presents a use case diagram that provides a high-level overview of the functionality of our serious game.

Following interviews with stakeholders, observation of rehabilitation sessions, and consideration for restrictions, the therapy to be transferred into the serious game was selected.

The standing human posture is innately unstable. The center of mass (COM) of the body is located approximately five-ninths of the body height from the ground with a narrow base of support (BOS). The active control of body alignment requires maintaining balance within the BOS. Activities of daily living (ADL) require control of the position of the COM over the BOS. A lack of skills or inappropriate skills for this control can result in the risk of falls and/or diminished quality of life.

Exercises for this type of active control include functional strengthening, balance activities to improve tone, and spatial orientation during postural control. Therapy also aims to strengthen the muscles of the neck, back, and upper limbs, which are used for balance and coordinating the upper limbs with the visual environment. In a standard therapy session, the following exercises are performed [32]:

- To start the therapy, the patient is asked to stand up (with his/her own technical aids if needed) from the sitting position.
- While standing, the patient performs COM movements for five minutes, using large and coordinated movements to displace the COM with speed, safety, and balance.
- In the same position, the patient performs 20 to 30 repetitions of forward-reaching exercises toward an object.
- In the same position, the patient performs 20 to 30 repetitions of left side-reaching exercises toward an object.

The same reaching exercises are then performed for the right side. The physiotherapist can adjust the amount of exercise according to the subject’s level of physical function to provide the optimal amount of exercise for each subject in accordance with the subject’s weekly tolerance for management of fatigue. Each participant has his/her exercise program updated or revised every week to improve his/her abilities in range of balance, motion, speed, and functional strengthening for postural control.

### Interaction mechanism

As described before, the development of a serious game is structured as three iterations. The goal of the first iteration is the definition of the interaction mechanism, which means determining how we can capture the therapy in an interaction system. The four framework activities within this iteration were performed as described below.

We designed a project management support form for agile planning and control. Table 3 represents an example of the use of this form for the interaction mechanism iteration.

**Table 3:** Planning and control tasks for the first iteration.

Framework activity	Task	Description
Modeling	Define the interaction mechanism	Analyze the selected therapy to define the interaction mechanism requirements.
Construction	Analyze tracking devices	Validate the suitability of existing devices to track the user’s hand.
	Select a tracking device	Select the most suitable tracking device. The selected device must be approved by the physiotherapists.
Validation	Validate and adapt the selected device	Validate the selected tracking device and adapt it to the interaction mechanism.

The objective of the first modeling task was the definition of the interaction mechanism requirements. This was accomplished by observing the standard therapy in real sessions. Engineers and therapists then held a series of meetings in order to define the characteristics of the interaction mechanism based on the engineers’ observations and the therapists’ knowledge. The following requirements were established for the interaction mechanism:

- R1. The tracking device must be able to detect the position of the patient’s hand at any moment to support the selected therapy.
- R2. Patients must be able to start the tracking with a simple gesture because they have difficulty holding physical devices.
- R3. The selected device must be noninvasive and affordable because of budget limitations.

In order to validate the suitability of existing motion-based capture devices, we identified the desirable features that they must support. We analyzed several different possibilities, as shown in Table 4.

**Table 4:** Motion-based device analysis.

Device	Noninvasive	Hand tracking	Motion detection	Cost
WebCam + markers			X	X
Eyetoy	X		X	X
Wiimote		X	X	X
Kinect	X	X	X	X

The results of this analysis led us to reject both the Wiimote and the combination of a webcam and markers because they are invasive for patients who have difficulty holding physical devices. The Eyetoy was also rejected because it neither tracks hand position nor detects the user when there is no motion. Other systems were rejected because they were outside the target budget. Finally, the Kinect RGBD device was selected as the best option because it fulfilled all the requirements. The selection of the Kinect device as a main reusable component was a way to save construction time and focus our efforts on the validation activity.

To complete the activities for interaction mechanism development, the only remaining task was to validate the suitability of the Kinect for the selected therapy. This was accomplished in two phases:

1. Recording the physiotherapist performing the therapy while we acquired ground-truth data for the hand position in each frame and compared the Kinect hand tracking positions with the recorded ground-truth.
2. Test the Kinect with real patients in a therapy session.

The comparison results in the first phase were accepted by the therapists. Therefore, Kinect hand tracking was deemed appropriate as an interaction mechanism for the selected therapy.

For the second validation phase, after testing the Kinect with real patients in a therapy session, we determined that the Kinect initialization procedure was not valid because Kinect initialization requires users to wave with one hand, which was not possible for some patients. Thus, a second iteration was needed. We applied the four model activities again for interaction mechanism development.

The result of the planning activity was the decision to construct a new tracking module adapted to our patients’ characteristics. The following requirement was added to the initial requirement list:

- R4. Before starting the game, the users must place their hand in a predefined position on the screen in order to start the hand tracking procedure.

**Fig 4:**
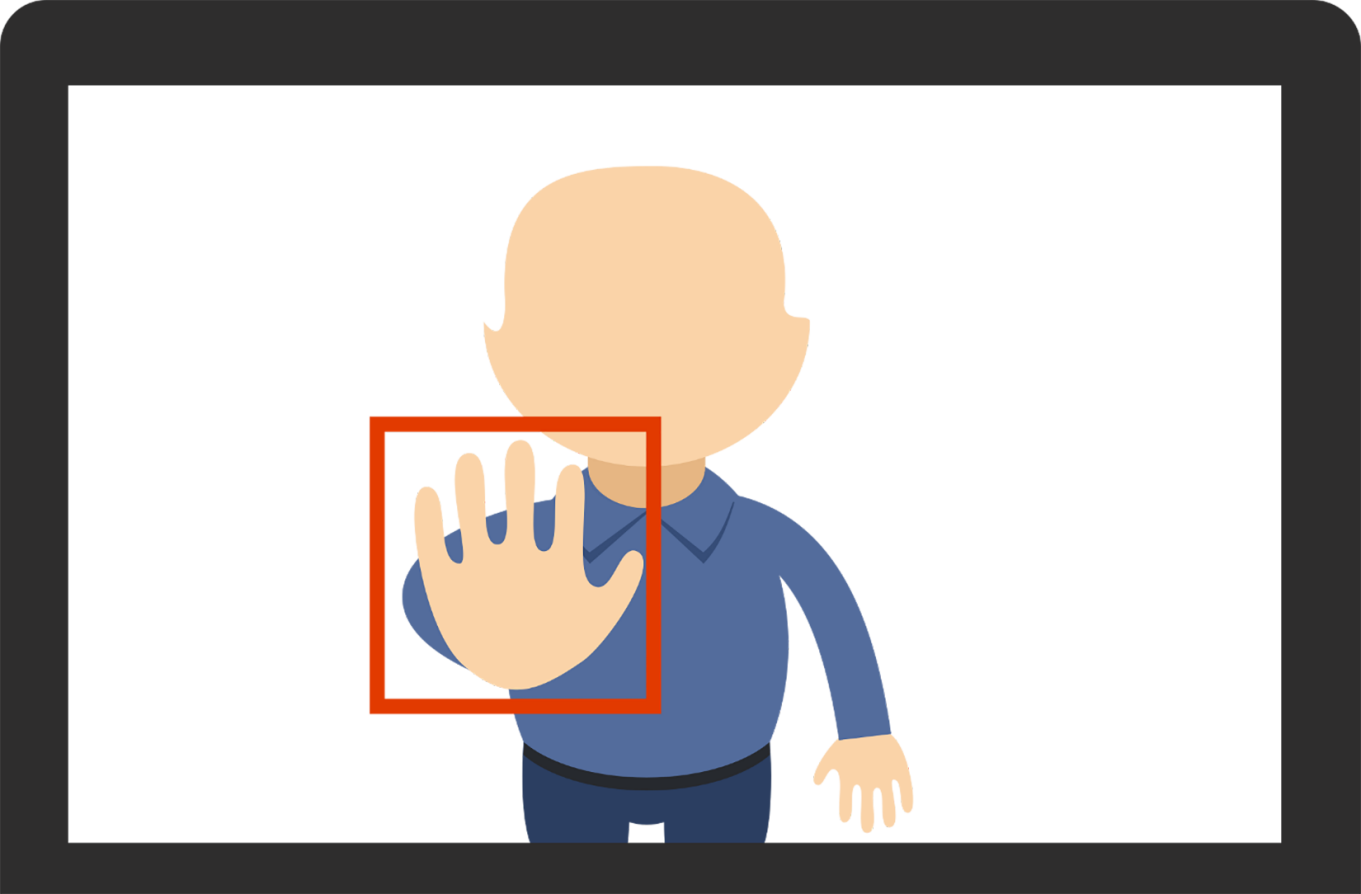
Tracking module interface.

To facilitate the new requirement, we designed the user interface to start with the hand tracking screen. As shown in Fig 4, we decided to design this interface by providing a square on the center of the screen where the users must place their hand in order to start the tracking procedure.

During the construction activity, we developed the previously designed module using the C++ programming language and the OpenCV computer vision library. Unit testing was performed in our lab to ensure the consistency of the developed software module. Finally, the initialization process was validated with real patients to prove that they were able to initiate the hand tracking procedure correctly.

The final result of the interaction mechanism iteration was a tracking module that would be integrated with the interaction module developed in the second iteration, as described in the following section.

### Interactive elements

The second iteration focuses on the design of interaction elements that force patients to perform the therapy correctly. In this case, that meant interacting with objects that could not be reached without moving the COM beyond the BOS. Just as for the first iteration described in the previous section, we follow the four framework activities for the design of the interactive elements. In this section, we only describe the most relevant activities and tasks.

From therapy observations, information collected from physiotherapist meetings, and a survey of existing hand-reaching interaction elements from interactive rehabilitation vision-based systems [4], the result of the modeling activity was the addition of two new requirements regarding the interactive elements:

- R5. Patients must interact with objects that cannot be reached without moving the COM beyond the BOS.
- R6. Patients must see themselves on the screen, as in a mirror, so that their position relative to the interactive objects is always known.

Based on these requirements, we defined a set of configuration parameters to customize the interaction and adapt it to different patients. The tunable conditions, which would become a part of the “*Configure game*” use case identified in the project initiation activity, are listed in Table 5.

**Table 5:** Conditions for the *“Configure game”* use case.

Design	Rationale
Number of elements to interact with. Position of each interaction element.	Definition of different levels in the game.
Distance between the user and the screen.	The larger the distance from the screen, the larger the required COM change.

**Fig 5:**
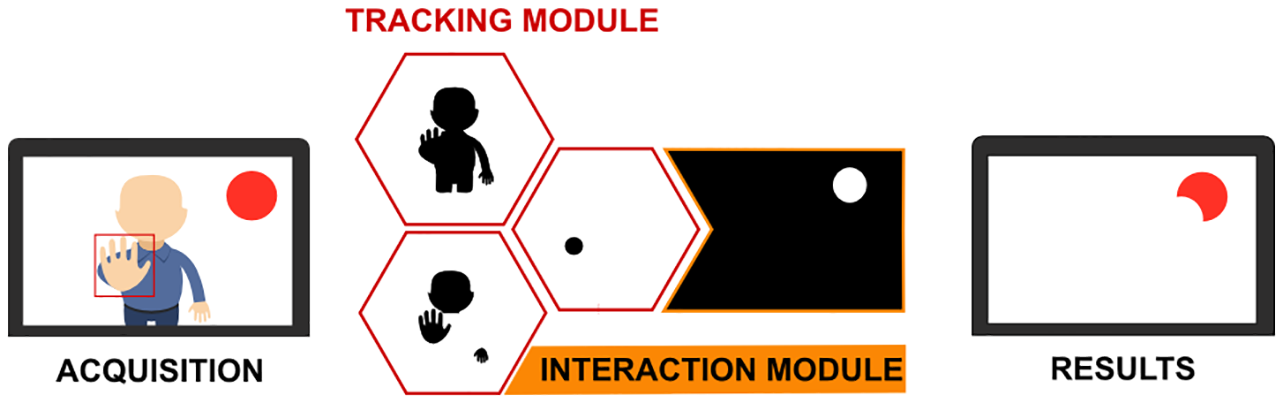
Interaction module.

With this design solution, we could start on the construction of the interaction module that would permit the user to interact with various objects. This module was integrated with the tracking module developed in the previous iteration (see Fig 5).

The validation of the interaction module was performed with real users, who tested different configurations of the tunable conditions proposed by the physiotherapists. The physiotherapists designed a set of interaction patterns by changing the number and positions of the interaction objects. These patterns were used to validate that the rehabilitation therapy was successfully transferred into the system and that the system could adapt the interaction based on the needs of different users. Additionally, different levels of difficulty were tested by changing the distance between the user and the camera in combination with the designed patterns.

### Serious game

The goal of the final iteration is the design, construction, and validation of a serious game that incorporates the physiotherapists’ recommendations and the desirable features for rehabilitation in serious games.

The result of the modeling activity was a more detailed requirement specification for the various use cases to be implemented (see Fig 3). This final specification is contained in Table 6.

**Table 6:** Requirements for the serious game.

Use case	Requirement
Create new user profile	R7. Patients must have a profile where their last-played game conditions are stored.
Configure game	R8. The therapist can set a time limit for each session. R9. The therapist can customize how long a player must be in contact with an element to erase that object. R10. Tunable conditions of interaction objects must be accessible from serious game configuration interface.
Play	R11. Each game action has corresponding visual and auditory feedback. R12. A visual object must be projected on the patient’s hand.
Validate therapy	R13. Patient performance must be stored for each game. R14. The therapist must be able to access patient information during the clinical study time period.

Based on these requirements, the proposed solution was the construction of a prototype that allowed patients to interact with colored circles that must be deleted by touching them as quickly as possible. During the validation of this prototype, we noticed that users were not motivated to play the proposed game. Therefore, the design of the serious game was enhanced with a new functionality that allows one to change the appearance of the objects to use images that increase motivation by displaying themes of particular interest to each patient.

The serious game was structured as four software modules that correspond to the four use case functionalities: user profile creation, game configuration, playing module, and data storage. The final module incorporates the interaction module constructed in the previous iteration.

**Fig 6:**
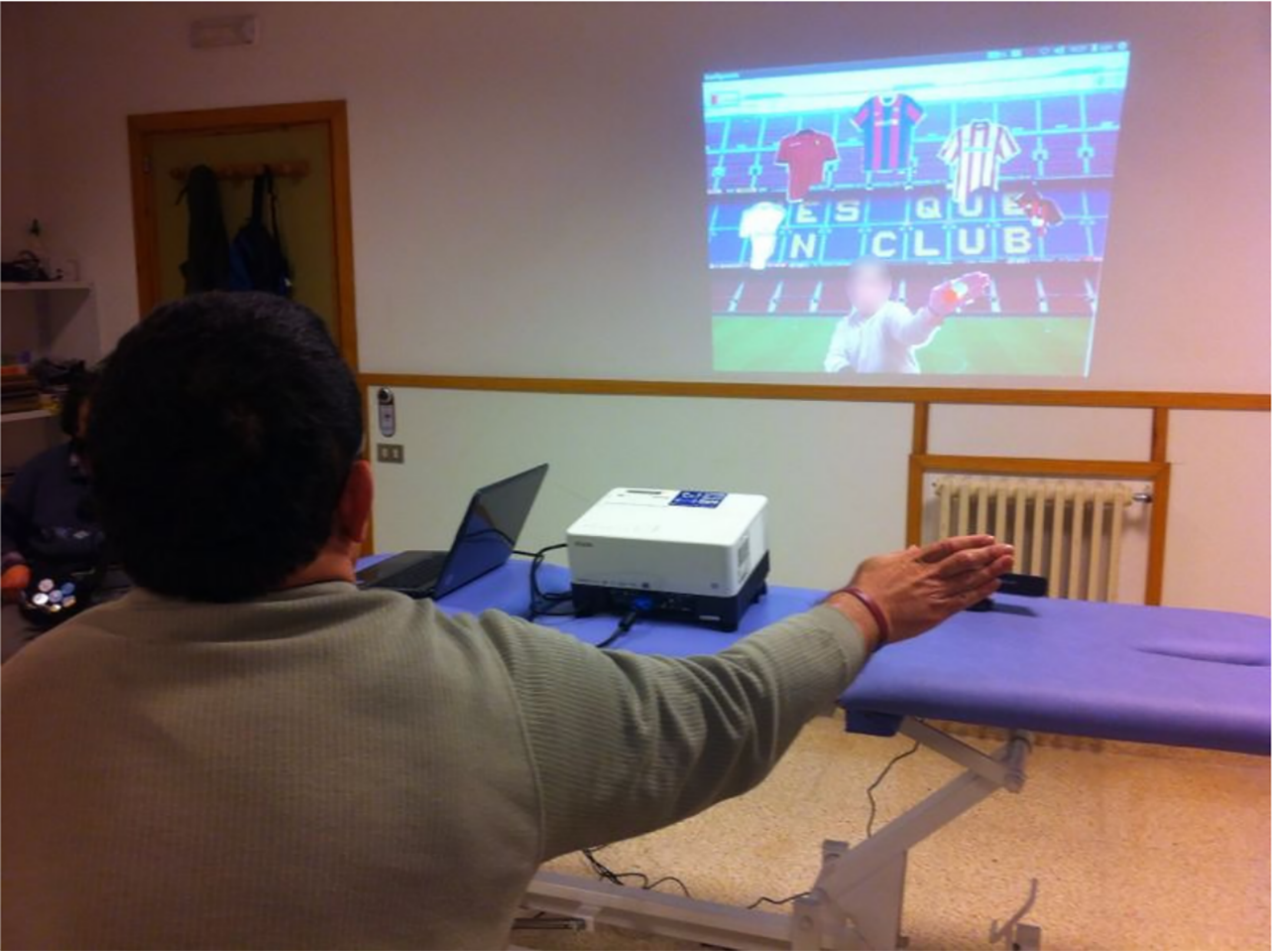
Therapy session.

The validation activity in this iteration simulated a therapy session. Eight patients performed the rehabilitation activity defined by the therapist. It was demonstrated that with interaction objects related to patient interests, patients performed the rehabilitation activity 13.5% (standard deviation of 4.3%) faster than when the objects did not represent such interests. Fig 6 shows a patient in a therapy session.

### Clinical study

As described above, the final activity of the framework is aimed at quantifying rehabilitation improvement. This activity consists of defining the experiment, the participants, and the measurements. These parameters were defined by the therapists.

The experiment consisted of a 24-week therapy period using the serious game, where patients were pre- and post-assessed before and after the therapy period. The participants played the game for at least 20 min. The number of repetitions varied according to participant tolerance and physiotherapist recommendations to better manage fatigue.

The participants were nine adults (seven males and two females), with a mean age of 37 years (range 27 to 57 years). All of them were diagnosed with CP and had limited voluntary motor control of one or both arms and legs, and of the trunk. The participants were recruited from the ASPACE in the Balearic Islands.

The measurements used to objectively assess balance were the following:

- Berg Balance Scale (BBS)
- Functional Reach Test (FRT, included in the BBD)
- Tinetti Balance Test (TTS)

According to the BBS, the assessment results demonstrated a significant functional improvement (p = 0.002) when comparing the pre- (29.5 ± 3.9) and post-assessment (34.1 ± 2.2) results. A comparison of functional balance revealed significant differences in the FRT before and after intervention (right upper limb (pre- (8.6 ± 1.4) and post-assessment (10.1 ± 2.0), p = 0.007) and left upper limb (pre- (8.3±2.0) and post-assessment (10.1 ± 3.7)), p = 0.052)). Finally, a significant difference between the pre- and post-assessment scores for the TTS was observed at the end of the 24-week intervention period (Jaume-i-Capó, Martinez-Bueso et al. 2014). The average score rose from 16 to 21 points on a scale of 28 points (improvement from high fall risk to moderate fall risk). These findings demonstrate a significant improvement in balance and gait function scores, which are indicators of greater independence for our participating adults.

## Conclusion

In this paper, we introduced a new process framework that supports the development of serious games for motor rehabilitation therapy. The framework is based on two core concepts. First, solutions are built while considering the similarities between web applications and serious games, which differ from conventional software systems. Second, the activities of the framework also consider the similarities between a clinical trial for drug development and serious games for motor rehabilitation.

The result is PROGame, a two-dimensional process flow where the basic development activities (planning and control, modeling, construction, and validation) are structured into three increments. The final activity of the model is a clinical study aimed at demonstrating the suitability of the serious game for the target therapy.

In order to validate the new framework, we applied it to the development of a serious game for the improvement of the balance and postural control of adults with CP. The serious game has been supported through two technological contracts and is currently being exploited by two organizations, which is a strong indication of system viability.

The application of PROGame has made us realize that with an organized, but flexible approach, it is easy to manage many emergent issues that affect the development of a serious game. We believe that the most important benefit of the model is a common process vision shared between the team members, which improves communication and facilitates task completion. Furthermore, the development results of this project have been supported by two technological contracts [14,15]. This validates the proposed framework because the serious game is being exploited by two organizations.

Of course, more work is needed to improve the model. We believe that PROGame provides the basis for a process improvement initiative by our team. Future work will be devoted to applying the model to new development projects as we analyze the suitability of the different activities to identify potential shortcomings and improvement opportunities, as well as to adapt it to the various scenarios of each project.
